# Supplementary material for: Non-classicality of the molecular vibrations assisting exciton energy transfer at room temperature
Source: Nat Commun. 2014 Jan 9;5:3012. doi: 10.1038/ncomms4012 (PMC3896760; doi:10.1038/ncomms4012)
Supplement: Supplementary Information — Supplementary Figures S1-S4 and Supplementary Notes 1-2 [file ncomms4012-s1.pdf]

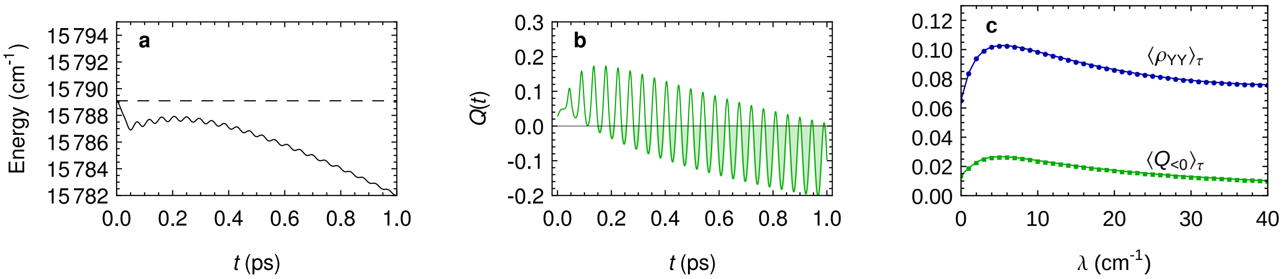


**Supplementary Figure S1**. The LHCII complex of green plants contains weakly electronically coupled dimers whose energy gaps are resonant with vibrational modes. Here we present results from the Chl$_{(b601)}$-Chl$_{(a602)}$ pair with electronic parameters[49] $\varepsilon_{(b601)} = 15764$ cm$^{-1}$, $\varepsilon_{(a602)} = 15103$ cm$^{-1}$, $V = -47.1$ cm$^{-1}$ and resulting $\Delta E = 667.7$ cm$^{-1}$. A vibrational mode of frequency $\omega_{\rm vib} = 742.0$ cm$^{-1}$ is close to this energy gap and each chromophore couples to this mode with strength $g = \omega_{\rm vib}\sqrt{0.03942} =$ 147.3 cm$^{-1}$, as obtained from ref. 36. The thermal background is characterized by $\lambda = 37$ cm$^{-1}$, $\Omega_{\rm c} = 30$ cm$^{-1}$ as in ref. 36. The strength of system-bath interactions $\sqrt{\lambda\Omega_{\rm c}}$ is smaller than in PE545 and therefore this dimer lies closer to the regime of coherent evolution of the exciton-vibration system, where the dissipation energy into the bath is transiently prevented. Shown here are **(a)** the energy of the exciton-vibration system and **(c)** the Mandel $Q$ parameter of the vibrational mode for $\lambda = 37$ cm$^{-1}$ (shaded regions denote times of non-classicality) and **(c)** averages over timescale $\tau \approx 0.4$ ps as a function of reorganization energy.

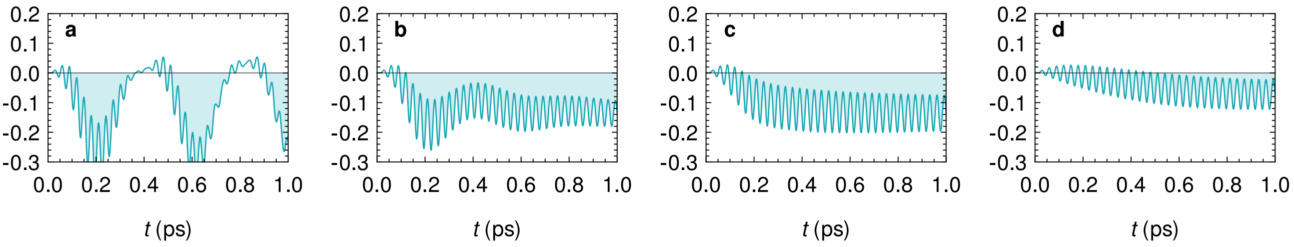


**Supplementary Figure S2**. **Non-classicality and modulations of adjacent phonon number occupations** The Mandel $Q$ parameter is a well established measure of the non-classicality of a quantized single mode bosonic field[37]. Its negativity (sub-Poissonianity) necessarily implies that there is no classical description for the field statistics in terms of a positive distribution function - for example the Glauber-Sudarshan $P$ representation will exhibit regions of negative density. Characterising the full phonon number distribution function $P(n)$ is not necessary to verify the type of non-classicality exhibited by these vibrational modes. Modulations of adjacent phonon number occupation as quantified by negative $B_n = (n+1)P(n-1)P(n+1) - nP(n)^2$, a criteria introduced by Klyshko[48], guarantees negative regions of a quasi-probability distribution and is a potentially an experimentally accessible quantity. The non-classicality indicated by sub-Poissonian statistics is confirmed by negative $B_1 = 2P(0)P(2) - P(1)^2$. This arises from selective population of the $P(1)$ level of the relative displacement vibrational mode. Here we present the Klyshko criterion $B_1$ for **(a)** $\lambda = 0$ cm$^{-1}$,**(b)** $\lambda = 6$ cm$^{-1}$, **(c)** $\lambda = 20$ cm$^{-1}$ and **(d)** $\lambda = 110$ cm$^{-1}$. Shaded regions denote times of non-classicality.

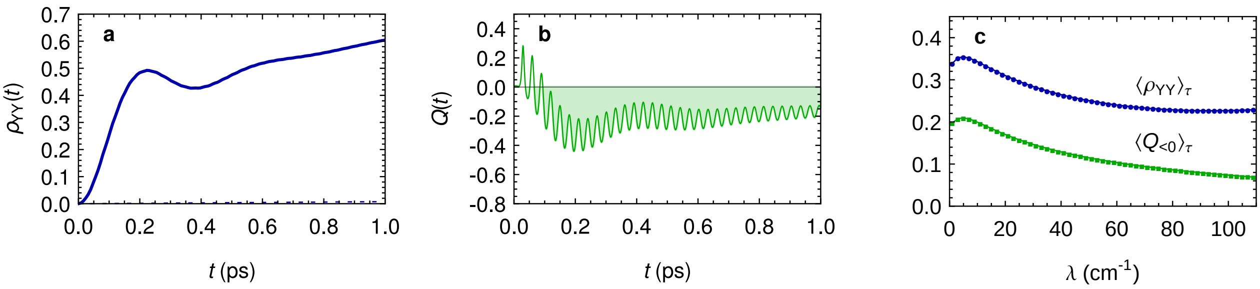


**Supplementary Figure S3**. **Damping of vibrational modes.** In the main text the quantized vibrational modes are treated as undamped because the timescale of their relaxation ($\sim$ 1 ps) is long with respect the timescale of evolution of the electronic system $\tau$. Here we damp vibrational modes by coupling them to a Markovian bath to demonstrate that our results and conclusions remain unchanged. The dissipator appearing in the hierarchical expansion is $\mathcal{D}(\varrho) = \sum_i \gamma_i(\bar{N}+1)\left(b_i\varrho b_i^\dagger - \frac{1}{2}\left\{b^\dagger b, \varrho\right\}\right) + \gamma_i \bar{N}\left(b_i^\dagger \varrho b_i - \frac{1}{2}\left\{bb^\dagger, \varrho\right\}\right)$ , where $\bar{N} = (e^{\beta\omega}-1)^{-1}$ is the mean thermal occupation of the mode. This causes an otherwise isolated mode to relax towards the thermal state $\varrho_{\mathrm{vib}}^{\mathrm{th}} = \sum_n P_{\mathrm{th}}(n)\left|n\right\rangle\left\langle n\right|$, $(P_{\mathrm{th}}(n) = (1-e^{-\beta\omega})e^{-\beta\omega n})$ on a time-scale $\gamma^{-1}$. **(a)** The evolution of $\rho_{YY}(t)$ and **(b)** the Mandel parameter $Q(t)$ when the vibrational mode is damped with strength $\gamma = (1\text{ ps})^{-1}$ for $\lambda = 6$ cm$^{-1}$. Shaded region denotes times of non-classicality. These should be compared with Fig. 4a and Fig. 5a in the main text. Other figures not shown here are also comparable for $t$ <1ps. **(c)** shows the time integrated averages as a function of reorganization energy which compares with Fig. 6 of the main text.

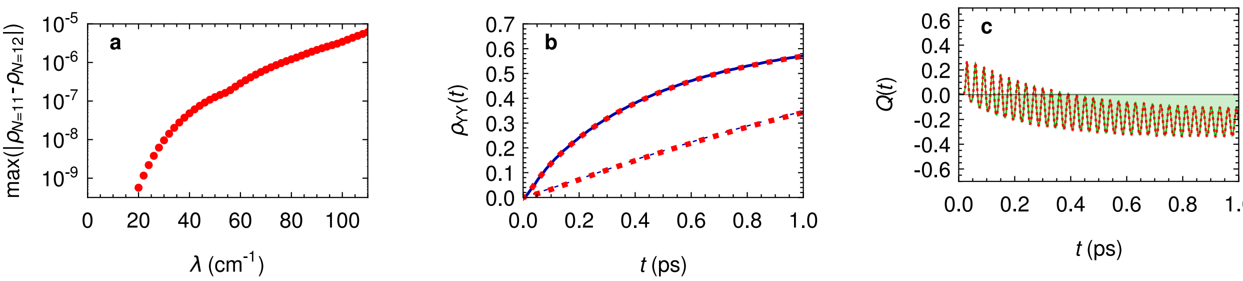


**Supplementary Figure S4**. **Numerical convergence.** A sufficient number of tiers of the hierarchy are needed to produce converged dynamics. Here we used $N = 11$ and found that the maximum differences in density matrix elements between $N = 11$ and 12 are at most $\sim 10^{-5}$ and generally substantially less. The Markovian truncation scheme employed in the hierarchical expansion of exciton-vibration dynamics negates the need for low temperature correction terms ($k > 0$) for $\beta\Omega_c < 1$ (for the studied systems $\beta\Omega_c \leq 0.47$). Here present results in PE545 with demonstrate convergence with respect to both $N$ and $K$. **(a)** Maximum differences in the system density matrix for the 1 ps time interval considered against reorganization energy $\lambda$, **(b)** $\rho_{YY}(t)$ with (solid) and without (dashed) vibrational mode for $\lambda = 100$ cm$^{-1}$ and **(c)**, the Mandel $Q$ of the vibrational mode. Shaded regions denote times of non-classicality. Red squares are results with a single temperature correction term included ($K = 1$).

**Supplementary Note 1: Hierarchical expansion of exciton-vibration dynamics**

Here we provide details of the model for a system consisting of $N$ electronic degrees of freedom each interacting with a quantized vibrational mode and the method used to accurately calculate the dynamics of these exciton-vibration systems. The system Hamiltonian $H_{\rm S} = H_{\rm ex} \otimes \mathbb{1}_{\rm vib} + \mathbb{1}_{\rm ex} \otimes H_{\rm vib} + H_{\text{ex-vib}}$, is defined by

$$H_{\rm ex} = \sum_{i=1}^{N} \varepsilon_i \sigma_i^+ \sigma_i^- + \sum_{i,j\leq i}^{N} V_{ij}(\sigma_i^+ \sigma_j^- + \sigma_j^+ \sigma_i^-) \ , \tag{S1}$$

$$H_{\rm vib} = \sum_{i=1}^{N} \omega_{\rm vib}^i b_i^\dagger b_i \tag{S2}$$

and

$$H_{\text{ex-vib}} = \sum_i g_i \sigma_i^+ \sigma_i^- \otimes (b_i^\dagger + b_i) \ . \tag{S3}$$

Here, the operators $\sigma_i^\pm$ create/annihilate electronic excitations at site $i$, while $b_i^\dagger$ ($b_i$) create (annihilate) phonons associated with the vibrational mode of site $i$. $\varepsilon_i$ denotes the excitation energies and $V_{ij}$ the strength of interactions between sites. $\omega_{\rm vib}^i$ is the frequency of the vibrational mode at site $i$ and $g_i = \sqrt{S_i}\omega_{\rm vib}^i$ the coupling of the mode to its site. $S_i$ is the Huang-Rhys factor or mean number of phonons in the 'polaron cloud' formed when the mode is fully displaced. The the high energy modes considered here, we expand the quantized vibrational modes of the system in the basis of single phonon (Fock) states (*e.g.* $b_i = \sum_n^M (n+1)^{1/2} |n\rangle \langle n+1|$) truncating at $M$ phonons. This system is then coupled to a continuum of bosonic modes (the thermal background) $H_{\rm B} = \sum_{\mathbf{k}} \omega_{\mathbf{k}} b_{\mathbf{k}}^\dagger b_{\mathbf{k}}$, with an interaction

$$H_{\rm I} = \sum_{\mathbf{k}} \sum_i g_{\mathbf{k},i}(\sigma_i^+ \sigma_i^- \otimes \mathbb{1}_{\rm vib})(b_{\mathbf{k}}^\dagger + b_{\mathbf{k}}) \ , \tag{S4}$$

such that a mode with frequency $\omega_{\mathbf{k}}$ is linearly coupled to the electronic excitation at site $i$ with a strength $g_{\mathbf{k},\mathbf{i}}$ as specified by a spectral density $J_i(\omega) = \pi \sum_{\mathbf{k}} |g_{\mathbf{k},\mathbf{i}}|^2 \delta(\omega - \omega_{\mathbf{k}})$. We assume the bath is spatially uncorrelated and identical for each site.

An infinite hierarchy of coupled differential equations[68–70] is used to express the dynamics of the exciton-vibration system density matrix $\rho_{\rm S}(t)$. These read as,

$$\begin{aligned}
\partial_t \tilde{\rho}_{\boldsymbol{n}}(t) = {} & -i/\hbar[H_{\rm S}, \tilde{\rho}_{\boldsymbol{n}}(t)] + \mathcal{D}(\tilde{\rho}_{\boldsymbol{n}}(t)) - \sum_{i=1}^{N}\sum_{k=0}^{K} n_{ik} v_{ik} \tilde{\rho}_{\boldsymbol{n}}(t) \\
& -i \sum_{i=1}^{N} \left( \sum_{k=1}^{\infty} \frac{c_{ik}}{\nu_{ik}} - \sum_{k=1}^{K} \frac{c_{ik}}{\nu_{ik}} \right) [Q_i, [Q_i, \tilde{\rho}_{\boldsymbol{n}}(t)]] \\
& - i \sum_{i=1}^{N}\sum_{k=0}^{K} \sqrt{(n_{ik}+1)|c_{ik}|} \left[ Q_i, \ \tilde{\rho}_{\boldsymbol{n}_{\boldsymbol{ik}}^+} \right] \\
& - i \sum_{i=1}^{N}\sum_{k=0}^{K} \sqrt{n_{ik}/|c_{ik}|} \left( c_{ik}\, Q_i \tilde{\rho}_{\boldsymbol{n}_{\boldsymbol{ik}}^-} - c_{ik}^*\, \tilde{\rho}_{\boldsymbol{n}_{\boldsymbol{ik}}^-} Q_i \right) \ .
\end{aligned} \tag{S5}$$

for the case of a Drude spectral density and in a rescaled formulation[70] which admits their efficient adaptive time-step numerical integration. Operators $\tilde{\rho}_{\boldsymbol{n}}$ are indexed by the multi-index $\boldsymbol{n}$ which has entries $n_{ik}$ and $n = \sum_{ik} n_{ik}$. $\boldsymbol{n}_{\boldsymbol{ik}}^\pm$ is $\boldsymbol{n}$ with entry $n_{jk} \to n_{jk} \pm 1$. The reduced density matrix of the system $\rho_S$ is given by the operator $\tilde{\rho}_{\mathbf{0}}$. The coefficients $\nu_{ik}$ and $c_{ik}$ are the Matsubara frequencies and coefficients appearing in the (truncated) exponential decomposition $\sum_{k=0}^{K} c_{ik} e^{-\nu_{ik} t}$ of the bath correlation function applied in ref. 69. In the above terms $k > K$ are truncated with a Markovian approximation scheme[69]. This results in accurate dynamics for $K = 0$ at high temperatures ($\beta\Omega_c < 1$). In the present exciton-vibration case the system-bath coupling operator takes the form $Q_i = \sigma_i^+ \sigma_i^- \otimes \mathbb{1}_{\rm vib}$. The dissipator $\mathcal{D}(\tilde{\rho}_{\boldsymbol{n}}(t))$ can phenomenologically describe other processes such as damping of the vibrational modes.

**Supplementary Note 2: Non-classicality Quasi-probabilities for quantized vibrations**

The non-classicality of a quantum state is unambiguously demonstrated by negativities in its description as a quasi-probability distribution. Here we calculate a regularized version of the $P$-representation

$$P_w(\alpha) = \frac{1}{\pi^2} \int \mathrm{d}^2\xi \; e^{\alpha\xi^* - \alpha^*\xi} \chi(\xi) \Omega_w(\xi) \;, \tag{S6}$$

where $\chi(\xi)$ is the characteristic function $\langle e^{\xi b^\dagger - \xi^* b} \rangle e^{|\xi|^2/2}$ of the state which is reconstructed from elements of the density matrix in the truncated Fock basis $\langle m| \varrho_{\mathrm{vib}} |n\rangle$. The non-classicality filter $\Omega_w(\xi)$ must satisfy the following conditions[39]:

**C1:** $\Omega_w(\xi) e^{|\xi|^2/2}$ is integrable for all positive $w$.

**C2:** $\Omega_w(\xi)$ has non-negative Fourier transform.

**C3:** $\Omega_w(0) = 1$ and $\lim_{w\to\infty} = 1$ for all $\xi$.

Consequently, any negativities displayed by the regularized $P_w(\alpha)$ with finite width imply the negativity of $P_{w\to\infty}(\alpha)$ (the usual $P$-representation) and therefore that the state is non-classical. We employ a simple a two-dimensional triangular filter, $\Omega_w(\xi' + i\xi'') = \mathrm{tri}(\xi'/w)\mathrm{tri}(\xi''/w)$ with $\mathrm{tri}(x) = 1 - |x|$ for $|x| < 1$ and $\mathrm{tri}(x) = 0$ otherwise. For any given non-classicality filter $\Omega_w(\xi)$ and filter width $w$, the regularized $P$-representation has the form

$$P_w(\alpha) = \sum_{m,n} \langle m| \varrho_{\mathrm{vib}} |n\rangle \left( \frac{1}{\pi^2} \int \mathrm{d}^2\xi \; e^{\alpha\xi^* - \alpha^*\xi} D_{nm}(\xi) e^{|\xi|^2/2} \Omega_w(\xi) \right) \;, \tag{S7}$$

with elements of the displacement operator in the Fock basis $D_{nm}(\xi) = \langle n| e^{\xi b^\dagger - \xi^* b} |m\rangle$ given by

$$D_{mn}(\alpha) = \begin{cases} \sqrt{\frac{n!}{m!}} (\alpha)^{m-n} e^{-|\alpha|^2/2} L_n^{m-n}(|\alpha|^2) & m \geq n \\ \sqrt{\frac{m!}{n!}} (-\alpha^*)^{n-m} e^{-|\alpha|^2/2} L_m^{n-m}(|\alpha|^2) & n \geq m \;, \end{cases} \tag{S8}$$

where $L_n^m(x)$ are the generalized Laguerre polynomials.
